# The Devil’s in the Detail: Accessibility of Specific Personal Memories Supports Rose-Tinted Self-Generalizations in Mental Health and Toxic Self-Generalizations in Clinical Depression

**DOI:** 10.1037/xge0000343

**Published:** 2017-06-29

**Authors:** Caitlin Hitchcock, Catrin Rees, Tim Dalgleish

**Affiliations:** Medical Research Council Cognition and Brain Sciences Unit, Cambridge, England; Medical Research Council Cognition and Brain Sciences Unit, Cambridge, England, and Cambridgeshire and Peterborough NHS Foundation Trust, Cambridge, England

**Keywords:** autobiographical memory, self-generalizations, rose-tinted beliefs, depression

## Abstract

Models of memory propose that separate systems underpin the storage and recollection of specific events from our past (e.g., *the first day at school*), and of the generic structure of our experiences (e.g., *how lonely I am*), and that interplay between these systems serves to optimize everyday cognition. Specifically, it is proposed that memories of discrete events help define the circumstances (boundary conditions) in which our generalized knowledge applies, thereby enhancing accuracy of memory-dependent cognitive processes. However, in the domain of self-judgment, cognition is systematically biased, with a robust self-enhancement bias characterizing healthy individuals and a negativity bias characterizing the clinically depressed. We hypothesized that self-enhancement effects in the mentally healthy may partly rest on an impaired ability for specific memories to set appropriate boundary conditions on positive self-generalizations, while the opposite may be true for self-referred negative traits in the depressed. To assess this, we asked healthy and depressed individuals to think about the applicability of a trait to themselves, then to recall a specific memory that was inconsistent with that trait which would therefore index a boundary condition for its applicability. Healthy individuals showed faster recall only for specific positive memories following negative trait evaluations, while depressed individuals demonstrated faster recall only of specific negative memories following positive trait evaluations—the pattern expected given the respective self-enhancement and negativity biases. Results suggest that specific memories may serve to delimit self-generalizations in biased ways, and thus support systemic biases in trait judgments characteristic of healthy and depressed individuals.

Our ability to navigate the challenges of daily life critically depends on our capacity to store and utilize memories of both specific events from our past (e.g., where we left our keys this morning) as well as the generic structure of our experiences (e.g., that we often feel lonely; McClelland, McNaughton, & O’Reilly, 1995). It has been proposed that these distinct mnemonic functions rely upon separable neural and cognitive systems. Tulving was among the first to advocate a functional distinction between episodic memories, or events experienced by oneself within a subjective space-time matrix, and semantic memories, or general knowledge about the world (e.g., Tulving & Donaldson, 1972), and variations on this broad multiple-memory-systems architecture are now ubiquitously endorsed (see Norman, 2013). There is also growing evidence that the brain’s hippocampal complex is involved in the fast encoding of specific episodes in ways that minimize interepisode interference, while slower neocortical systems extract overlapping information across multiple episodes in the construction of general semantic representations of the world (e.g., Kumaran & McClelland, 2012). Tulving (e.g., 1989, 2002) drew a further distinction within the semantic system between generic knowledge of the world (e.g., that cats purr) and what he termed semantic personal memories—general facts about oneself and one’s past (e.g., I had a mostly happy childhood).

Within autobiographical memory, the separability of neural and functional systems underpinning the processing of such semantic personal memories and of specific personal episodes is supported by neuropsychological case studies (e.g., Klein & Lax, 2010; Tulving, Schacter, McLachlan, & Moscovitch, 1988), cognitive science (Conway & Pleydell-Pearce, 2000; Renoult, Davidson, Palombo, Moscovitch, & Levine, 2012) and neuroimaging data (e.g., D’Argembeau & Salmon, 2012; Levine et al., 2004). A particularly fertile research landscape has been the interplay between semantic personal memories pertaining to one’s personal traits (e.g., I am generally helpful) and specific memories of behavioral episodes relevant to those traits, and in a series of experimental and neuropsychological studies, Klein and Loftus provide evidence that these two distinct forms of self-knowledge are independently utilized and represented (e.g., Klein, Loftus, & Sherman, 1993; see Klein et al., 2002, for a review).

One reason why personal semantic knowledge concerning one’s traits is of particular interest is because the degree of validity of such knowledge varies as a function of context. “Having blue eyes” is an invariant item of personal semantic information. ‘Being friendly,’ in contrast, even for the friendliest of people is likely to be somewhat context-dependent, coming with subjective terms and conditions attached (Mischel, 1969). In such cases where the applicability of a trait judgment is not entirely consistent across situations, the obvious processing advantages conferred by such generic semantic representations—that they are faster to access and utilize within decision contexts that often require split-second decisions, is mitigated by necessary reductions in their accuracy in any given situation. The question of how the memory system can be organized to maximize accuracy across situations therefore becomes pertinent (Klein et al., 2002). One potential solution recruits the independent store of specific behavioral episodes to mitigate the inaccuracies inherent in generic trait judgments by providing information from the past about the particular ‘boundary conditions’ under which such summary representations do and do not apply (e.g., situations when I am not friendly).

This putative solution gives rise to a counterintuitive prediction, that in decision situations where trait judgments about oneself are required, there should not only be activation of the personal semantic information relevant to that trait within the semantic memory store, but also concurrent activation within the episodic store of specific behavioral episodes that define the ‘boundary conditions’ within which the trait judgment is valid (Klein et al., 2002). Klein and colleagues termed this proposal the Scope Hypothesis. In an elegant series of memory priming experiments supporting this hypothesis they demonstrated that when participants were asked to reflect on the degree to which a given trait characteristic (e.g., *friendly*) was true of them, they were subsequently quicker to recall a specific personal memory of an episode when they had behaved in a manner that was *inconsistent* with that trait (e.g., a time when they had been unfriendly). This priming of inconsistent episodes was not present when participants were simply asked to define the trait word—a context that does not cause retrieval of a personal trait summary. Consistent with the broad rationale of priming studies whereby primed target material is rendered more accessible and/or faster to process, Klein et al. concluded that this faster access of specific memories (in this instance when primed by reflecting upon incongruent traits) indexed a temporarily greater accessibility of those episodes within the autobiographical memory system. This interpretation is in line with the proposed role of those specific memories in providing boundary conditions for the trait judgment. Interestingly, accessing personal trait information does not appear to prime memories of specific episodes *consistent* with that trait (e.g., Klein, Loftus, & Burton, 1989; Klein, & Loftus, 1990, 1993; Klein, Loftus, Trafton, & Fuhrman, 1992) in line with the notion that personal semantic information and episodic information are independently stored (see also, e.g., Klein, Loftus, & Kihlstrom, 1996, for supporting neuropsychological evidence).

This key theoretical notion—that accessing semantic or generic representations is linked to the access of representations of specific *inconsistent* information—is not limited to the domain of autobiographical memory. For example, in Schank’s (1980, 1982) seminal work on script-based learning, the scripts derived from repetitions within the learning context are also proposed to contain information pertaining to expectancy-violating experiences. Schema models of memory make similar claims. Bartlett’s (1932) original “schema with correction” model describes schematic representations that code regularities across experiences alongside specific deviations from those regularities. More recently, Graesser’s “schema pointer plus tag” model proposed generalized semantic representations stored along with tags linking to schema-inconsistent episodes that are retrieved whenever the schema is activated (e.g., Graesser, Gordon, & Sawyer, 1979). Similar conceptualizations are outlined in the domains of social judgment (e.g., Babey, Queller, & Klein’s, 1998, Summary-plus-Exception Model) and category learning (e.g., the Nosofsky, Palmeri, & McKinley, 1994, RULEX model).

A central tenet of the Scope Hypothesis (and of the family of similar theories in other domains) is that our overall *accuracy* in applying trait judgments is enhanced when the scope of more speedily retrieved but less detailed summary memories is delimited by situationally specific information that is slower to access but more precise (Klein et al., 2002). However, if the memory system is really organized to enhance accuracy then this raises an important question: Why are personal trait judgments so often systematically biased? This question is the focus of the present study. In particular, we consider this issue with respect to the positivity or self-enhancement bias (Brown, 1986; see Sedikides & Gregg, 2008; Taylor & Brown, 1988) characteristic of healthy participants when evaluating their own traits, and the negativity bias evident in similar evaluations in those suffering from clinical depression (see Beck, 2008; Dalgleish & Werner-Seidler, 2014; Gotlib & Joorman, 2010).

One of the more robust findings within social and personality psychology is that healthy individuals possess and express overly positive, rather than realistic, self-perceptions (for discussion see Taylor & Brown, 1988), judging positive traits to be far more characteristic of the self than negative attributes (Alicke, 1985; Brown, 1986). The unrealistic nature of these judgments is evident from the so-called Better-than-Average-Effect, where healthy individuals tend to rate positive traits as more descriptive of themselves relative to others, across the board (e.g., Alicke, Klotz, Breitenbecher, Yurak, & Vredenburg, 1995), and from the fact that self-evaluations are systematically more positive than those of independent observers (e.g., Lewinsohn, Mischel, Chaplin, & Barton, 1980). This self-related positivity effect extends beyond trait judgments, encompassing attributions (see Mezulis, Abramson, Hyde, & Hankin, 2004), and evaluations of the future (see Sharot, 2011). Of relevance here is that this overall bias also extends to the recollection of memories of specific events. Healthy individuals show better recall for information pertaining to personal success than to failure (Silverman, 1964), tend to recall their own task performance as more positive than it objectively was (Anderson, Brion, Moore, & Kennedy, 2012; Crary, 1966; Gramzow, Elliot, Asher, & McGregor, 2003), and are faster to retrieve autobiographical memories of positive relative to negative events (see Blaney, 1986; Rubin & Berntsen, 2003).

A very different pattern of effects is found in sufferers of clinical depression where unrealistic negative trait judgments are pervasive (Greenberg & Beck, 1989; Greenberg & Alloy, 1989). Indeed, modifying the generic representations that underpin such judgments and other forms of negative cognition, by shedding light on their unrealistic nature, is a central tenet of cognitive therapies to ameliorate depression (Beck, Rush, Shaw, & Emery,1979). As with the opposite pattern of positive biases in healthy participants, negative biases are also apparent when examining patterns of retrieval of specific behavioral episodes from memory in those with depression. A relative difficulty in retrieving specific memories generally is a feature of the disorder (Williams et al., 2007), with recollection of positive specific memories being particularly affected (Dalgleish & Werner-Seidler, 2014; Williams & Scott, 1988) and thus a target for selective therapeutic intervention (e.g., Dalgleish et al., 2013).^1^

How do the self-enhancement bias characteristic of healthy individuals and the negativity bias characteristic of those with clinical levels of depression relate to the theoretical notion that the different memory systems involved in making personal trait decisions are optimized for accuracy? One possibility is that the implementation of the Scope Hypothesis within those systems is itself biased—something that was not a focus of Klein et al.’s (2001, 2002) original studies. In those terms, the coactivation or priming of specific memories that are inconsistent with positive trait memories in healthy individuals, and with negative trait memories in those who are depressed, could be relatively weak. In turn, the priming of specific memories that are inconsistent with negative trait summaries in healthy individuals, and with positive trait summaries in depressed people, could be relatively strong. In Klein et al.’s (2002) terms, for the mentally healthy, specific negative memories would impose looser boundary conditions (as indexed by weaker priming) on the scope of positive summary trait information, whereas for those who are depressed, positive memories would impose looser boundary conditions on the scope of negative trait summaries, thus leading to systematic patterns of positive and negative trait bias, respectively.

In the present study we sought to investigate these two possibilities utilizing an adaptation of Klein et al.’s priming methodology (Klein, Cosmides, Tooby, & Chance., 2001) with currently healthy and never-depressed individuals, with currently clinically depressed individuals, and also with individuals in remission from recurrent depression. The reasoning behind including this latter group is that there is evidence that some of the pervasive cognitive biases that characterize acute depression, including impoverished specific memory access, and differential accessibility of negative trait information, continue to operate during periods of remission in those who experience recurrent episodes, serving as a vulnerability factor for relapse (e.g., Mackinger, Pachinger, Leibetseder, & Fartacek, 2000; Teasdale & Dent, 1987).

Our hypotheses were that for the healthy, never-depressed individuals, activation of negative trait generalizations would prime faster access to inconsistent specific positive memories (compared to Klein et al.’s, 2001, word definition control condition), but that this effect would be attenuated or absent in depressed individuals. In contrast, we hypothesized that activation of positive trait generalizations would prime access to inconsistent negative specific memories for depressed individuals, with an attenuation or absence of this effect in the never-depressed sample. We had no specific hypotheses about the direction of effects in remitted individuals as it was unclear a priori whether the hypothesized biases would be latent in a remitted sample (and thus only evident following some form of mood or cognitive activation manipulation; Debeer, Raes, Williams, & Hermans, 2011) or would be explicitly detectable (cf., Williams et al., 2007).

## Method

### Participants

The priming effect (*d* = 1.77) originally reported by Klein et al. (2001) for the contrast of interest was very large. A two-tailed sample size estimation, with α = .05 and power at 90%, based on this original priming effect size indicated that a minimum of 10 participants would be required to detect within-group effects. This would also provide sufficient power to detect any between-group interactions as these within-group effects were predicted to act in opposite directions to each other for the never-depressed and depressed groups. However, we conservatively aimed to recruit at least 50% more participants than the required number estimated by this calculation (i.e., 15 per group), as we were focusing on the difference in the size of this effect as a function of valence within each group.

Participants were recruited from the clinical and nonclinical volunteer panels at the MRC Cognition and Brain Sciences Unit. The sample comprised 27 (15 female) healthy individuals with no lifetime history of depression, and 35 (23 female) individuals who met criteria for a lifetime diagnosis of Major Depressive Disorder (MDD), 18 (12 female) of this latter group were currently in remission, and 17 (11 female) were currently experiencing a Major Depressive Episode. Depression status was determined by trained research staff using the Structured Clinical Interview for the *DSM–IV* (SCID; First, Spitzer, Gibbons, & Williams, 1996).

The sample was predominately Caucasian (95.2%) and had completed high school (74.2%). Approximately half of the participants were currently employed (48.4%). The mean age of participants was 41.06 years (*SD* = 17.00, range = 18–78 years). Eight currently depressed participants were receiving psychological treatment and 13 were receiving antidepressant medication. No individuals were receiving stimulant medication which may have influenced response times.

### Autobiographical Memory Priming Task

**Figure 1:**
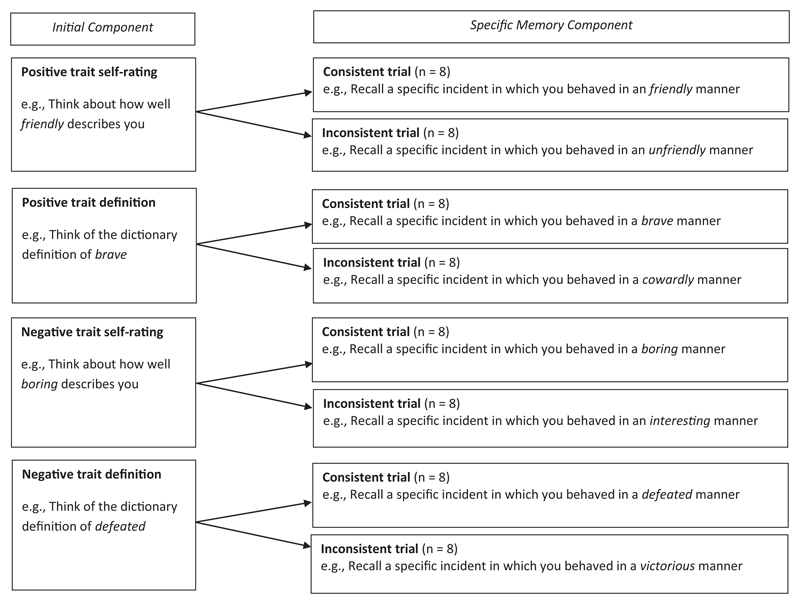
Design of the Autobiographical Memory Priming Task.

The Autobiographical Memory Priming Task (AMPT) was closely derived from that used by Klein et al. (2001). Each trial comprised an initial component—processing a trait adjective that is either positively or negatively valenced—followed by a second component—recalling a specific memory (see Figure 1). For the initial component, participants completed either (a) a trait self-rating task, in which they were asked to think about how well the presented trait adjective described them or (b) a definition task, in which they were asked to think about the “dictionary definition” for that trait characteristic. The definition task served as a control condition as it required the individual to access information about the characteristic in a manner that was not explicitly self-referential. In the second component, participants were asked to recall a specific autobiographical memory that either: a) involved behavior consistent with the trait characteristic (i.e., a consistent memory), or b) involved behavior inconsistent with the characteristic (i.e., an inconsistent memory). The dependent variable was the time taken for participants to recall these specific memories.

The AMPT therefore comprised eight conditions in a 2 (Initial Component: Self-Rating, Dictionary Definition Control) × 2 (Valence of the Initial Component: Positive, negative) × 2 (Consistency of the specific memory with the initial component cue: Consistent, inconsistent) design. Participants completed 8 trials per condition (totaling 64 trials; see Figure 1).

For the trait characteristic stimuli we selected 32 positive and 32 negative person-descriptive words from Dumas, Johnson, and Lynch (2002). The positivity or negativity of the characteristic was determined by likeableness and pleasantness ratings in the Dumas et al. corpus (see Dumas et al., 2002 for details of how ratings were obtained). Positive cues were significantly more likable (*M* = 4.97, *SD* = 0.56) than negative cues (*M* = 2.07, *SD* = 0.50), *t*(126) = 31.04, *p* < .001, *d* = 5.53. Positive cues (*M* = 755.74, *SD* = 59.29) were also more pleasant, (negative *M* = 223.42, *SD* = 63.63), *t*(78) = 38.73, *p* < .001, *d* = 8.77. To determine antonyms for each of these characteristics, we searched the characteristic in Roget’s Thesaurus (Davidson, 2006), and selected an antonym listed for the characteristic that was also included in the Dumas et al. (2002) wordlist to allow us to use the Dumas et al. ratings. The experimental stimuli were comparable between conditions, with no significant interactions between valence (i.e., positive or negative) and the task-component the word was used for (i.e., as a cue for the initial component or as a specific memory cue) in predicting Kuèera-Francis word frequency, *F*(1, 124) = 2.55, *p* = .11, $\eta_{p}^{2}$ = .02, meaningfulness, *F*(1, 88) = 0.59, *p* = .45, $\eta_{p}^{2}$ = .01, and familiarity, *F*(1, 124) = 0.03, *p* = .88, $\eta_{p}^{2}$ < .01, according to the MRC Psycholinguistic Database (Wilson, 1988). Cue words were randomly assigned to experimental condition between participants.

The AMPT was presented on a computer and programmed using E-Prime 2.0 software (Psychology Software Tools, 2012). Prior to beginning the test trials, on-screen instructions were presented for the initial component stating that participants would need to either think about how much the personality characteristic described them or to think about the dictionary definition for the word. For the second component, participants were instructed that they would need to recall a specific incident: “*an event that occurred on one particular time and lasted for less than a day,*” and an example was provided. Participants were informed that it was important that they immediately indicate when they had completed each component by pressing any computer key.

For each test trial, a short reminder of the task instructions and the relevant cue were simultaneously presented and remained on the screen until participants selected a key to show that they had completed that component. The time interval between screen presentation and pressing the key was recorded. The time it took for participants to press a key to indicate recall of a specific memory on the second component of the task was our dependent variable. Following completion of the initial component, a blank screen appeared for 1 second, and then the specific memory instructions and cue were presented. To remind participants of task instructions for the second component, after indicating that they had brought a memory to mind, a screen asked, “Was your memory of one particular time/one particular event?” If the participant indicated that it was not a specific incident, they were reminded that they needed to recall a specific incident. The reason for this was that with consistent prompting, depressed individuals are able to overcome their natural difficulty in accessing specific memories (Dalgleish et al., 2007). There was no significant difference in the proportions of initial declared nonspecific responses between the healthy (*M* = .04, *SD* = .06), depressed (*M* = .11, *SD* = .20), and remitted (*M* = .13, *SD* = .20) groups, *F* (2, 54) = 1.95, *p* = .152, $\eta_{p}^{2}$ = .07. After a 2-second intertrial interval the next trial was presented.

Prior to the 64 test trials, participants completed eight practice trials (one for each experimental condition) during which they were required to report their memories aloud to ensure that task instructions were understood. After completing the AMPT, participants were presented with each cue used in the experiment, and asked to rate the cue on a 9 point scale from 1 = ‘*extremely unlike me*’ to 9 ‘*extremely like me*’. These ratings allowed us to consider the effect of cue self-relevance on our experimental findings.

### Procedure

Ethics approval was obtained from the East of England committee of the NHS National Research Ethics Service. All participants provided informed consent. Participants were informed that they would be completing a number of tasks involving the processing of personal trait adjectives. Participants first completed the experimental task, followed by a short break and then they completed the cue word ratings. Participants next completed the Beck Depression Inventory- Second Edition (BDI-II), a gold-standard self-report measure of depression symptoms (Beck, Steer, & Brown, 1996). To allow us to assess whether verbal ability and fluency affected performance on the experimental tasks, participants also completed the Mill Hill Vocabulary Scale—Synonyms Test Form B (Raven & John Hugh Court, 1998), a measure of acquired verbal knowledge in which participants are required to select a synonym for 34 English words, and the Verbal Fluency Task, which required participants to retrieve as many words as possible in one minute that matched the given category (words begining with the letter *A* and types of animals; Tombaugh, Kozak, & Rees, 1999). Participants received an honorarium of £12 for their time.

## Results

The latencies to retrieve specific memories were transformed using a square root transformation (see supplemental materials for details) to remove the positive skew common to response time data (Bargh & Chartrand, 2014; Tabachnick & Fidell, 2001). Transformed data were therefore used in all analyses. Data were excluded from three participants in the never-depressed group, one in the remitted and one in the depressed group, due to noncompliance with the experimental protocol (see supplementary materials for details). The analyzed dataset therefore comprised 24 participants in the never-depressed group, 17 in the remitted group, and 16 in the depressed group.

### Sample Characteristics

As anticipated, the depressed group had higher BDI-II scores (descriptive statistics in Table 1) than the remitted, *p* < .001, *d* = 0.58, and never-depressed, *p* < .001, *d* = 2.91, groups, *F* (2, 54) = 38.74, *p* < .001, $\eta_{p}^{2}$ = .64. As further anticipated, the remitted group also demonstrated significantly more symptoms on the BDI-II than never-depressed participants, *p* = .009, *d* = 1.25. The mean BDI-II score indicated a ‘moderate’ level of symptom severity for the depressed group and a ‘minimal’ level of symptom severity for the never-depressed and remitted groups (Beck et al., 1996). In terms of the number of previously experienced depressive episodes, 12 of the currently depressed participants had experienced too many depressive episodes to count the number of distinct episodes (this represents one of the coding categories on the SCID; First et al., 1996). This was also the case for three of the remitted individuals. For those remitted participants able to identify the number of previous episodes, the mean number of episodes was 4.40 (*SD* = 3.11), consistent with the experience of recurrent depression. Age did differ between the never-depressed and remitted, *p* < .001, and depressed conditions, *p* < .001, *F* (2, 59) = 21.72, *p* < .001. Although age was not associated with performance on any of the outcome measures, *r*s (*n* = 62) < −.10, *p*s > .423, we repeated our key between-groups analyses with age included as a covariate and the results were unchanged (and in fact the effects were stronger). All groups were comparable in terms of scores on the Mill Hill Vocabulary test, *F* (2, 54) = 1.88, *p* = .163, $\eta_{p}^{2}$ = .05, and the Verbal Fluency Task *F* (2, 54) = 1.30, *p* = .280, $\eta_{p}^{2}$ = .07.

### Hypothesis Testing

**Figure 2:**
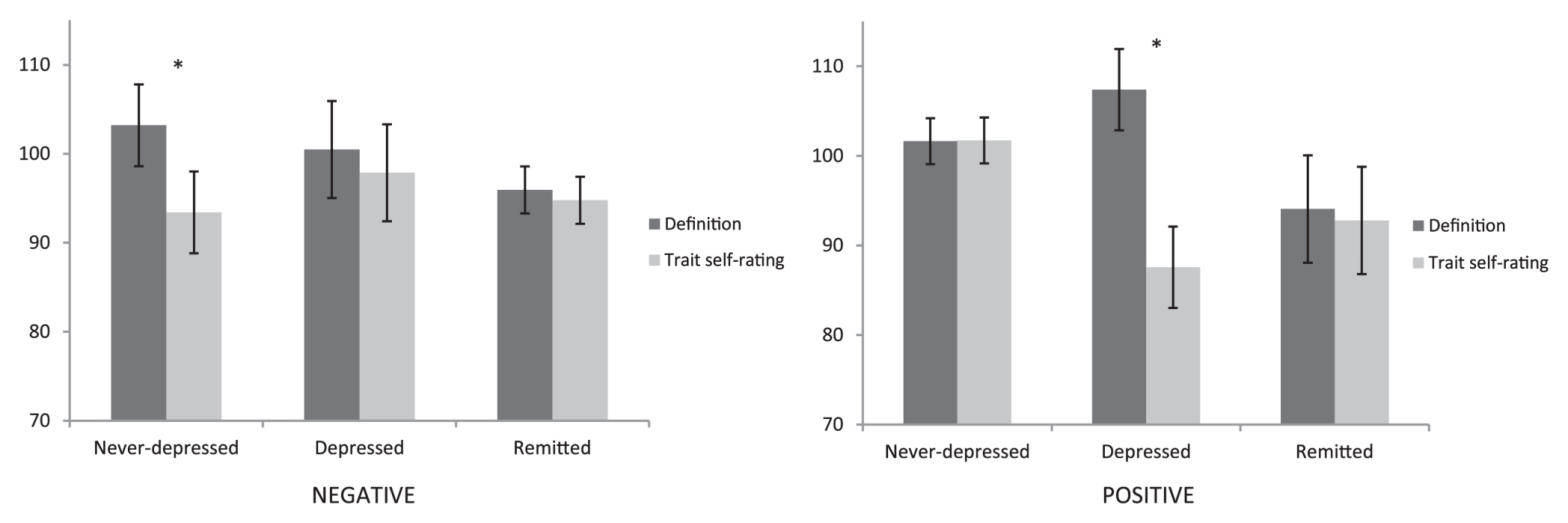
Mean (square root transformed) number of milliseconds to recall an inconsistent specific memory by valence of the trait and type of initial component for each group. Untransfomed data are presented in the supplementary materials. Note. * *p* < .05. Error bars are standard error of the mean.

Figure 2 presents the response times (square root transformed) for recall of an inconsistent specific memory across the different groups and task components. Complete response time data for recall of a specific memory across all of the different AMPT conditions is presented, by group, in supplementary Table 1. If the speed of access to a specific memory is an index of its capacity to delimit the scope of a preceding trait self-rating for which the memory is inconsistent, then we would expect response time on inconsistent memory trials to be shorter when the initial component involved a trait generalization self-rating (relative to the dictionary definition comparison condition) - the priming effect (Klein et al., 2001). As outlined in the Introduction, we had two key hypotheses. We expected that for the healthy, never-depressed group, this priming effect would be greater following negative trait self-ratings than for positive trait self-ratings. For the depressed group, we expected the opposite with the priming effect being greater following positive trait-self-ratings than following negative trait self-ratings. We had no a priori prediction for the remitted group.

We initially conducted an omnibus 3 (Group: Never-depressed, remitted, depressed) × 2 (Valence of the trait self-rating: Positive, negative) × 2 (Initial component: Self-rating, dictionary definition) × 2 (Consistency of the specific memory: Inconsistent, consistent) mixed-model ANOVA with time taken to recall a specific memory (square root transformed) as the dependent variable. We observed main effects of task, *F* (1, 54) = 4.17, *p* = .046, $\eta_{p}^{2}$ = .07, and consistency, *F* (1, 54) = 3.97, *p* = .051, $\eta_{p}^{2}$ = .07, and an interaction between task and consistency, *F* (1, 54) = 5.29, *p* = .025, $\eta_{p}^{2}$ = .09, all qualified by a significant four-way interaction, *F* (2, 54) = 5.38, *p* = .007, $\eta_{p}^{2}$ = .17, consistent with our specific predictions.^2^ None of the other main effects or interactions reached significance *F*s < 2.61, *p*s > .083.

We examined this four-way interaction between each pairing of the three groups. Performance on the AMPT was significantly different between never-depressed and depressed conditions, *F* (1, 39) = 9.74, *p* = .003, $\eta_{p}^{2}$ = .20, as expected. However the remitted group did not differ from either the never-depressed, *F* (1, 40) = 1.34, *p* = .253, $\eta_{p}^{2}$ = .03, or the depressed groups, *F* (1, 32) = 3.76, *p* = .061, $\eta_{p}^{2}$ = .11.

Having established that there was a significant differential effect across the depressed and never-depressed groups, we next examined each of these two groups separately to explore our specific hypotheses. For the never-depressed group, there was a significant 3-way interaction of Valence × Task × Consistency, *F* (1, 23) = 5.32, *p* = .031, $\eta_{p}^{2}$ = .19, consistent with the differential pattern of task performance across positive and negative trials that we predicted (see Figure 2). Breaking this effect down by valence, a Significant Task × Consistency interaction was observed for negative trials, *F* (1, 23) = 4.45, *p* = .046, $\eta_{p}^{2}$ = .13. In line with faster access to inconsistent specific memories delimiting the scope of the prior negative self-rated trait, response time on inconsistent trials was significantly shorter following a negative trait self-rating, relative to a definition of a negative trait, *t* (23) = 2.13, *p* = .044, *d* = 0.24 (see Figure 2). This difference was not evident on consistent trials, *t* (23) = 0.15, *p* = .882, *d* = 0.02. The Task × Consistency interaction was nonsignificant for positive trials, *F* < 1.

For the depressed group there was a near significant 3-way interaction of Valence × Task × Consistency, *F* (1, 15) = 4.28, *p* = .056, $\eta_{p}^{2}$ = .22. Deconstructing this by valence, we found a Significant Task × Consistency interaction for positive trials, *F* (1, 15) = 6.57, *p* = .022, $\eta_{p}^{2}$ = .31, but not for negative trials, *F* < 1. As predicted, response time on inconsistent trials was significantly shorter when following a self-rating of a positive trait than when following a dictionary definition of the trait, *t* (15) = 4.37, *p* = .001, *d* = 0.35. Again, this effect was not significant for consistent trials, *t* (15) = 1.69, *p* = .112, *d* = 0.15.

Although the pattern of performance in the remitted group was not significantly different to that of either of our other groups, we nevertheless examined the profile of task performance within this group. However, unlike the depressed and never-depressed groups, we found no support for a 3-way interaction of Valence × Task × Consistency, *F* < 1.

## Discussion

Klein and colleagues’ Scope Hypothesis (Klein et al., 2001, Klein, Cosmides, Tooby, & Chance, 2002) argues that when we retrieve personal semantic information (Tulving, 2002) that has context-dependent veridicality, memories of specific autobiographical episodes that represent exceptions to that information are primed such that the boundary conditions or ‘scope’ for when the semantic information is true, versus when it is invalid, can be delimited. For instance, a self-referent trait judgment such as “*Am I friendly*?” would involve retrieval of generic personal semantic information about my overall degree of friendliness, paralleled by priming of memories of specific episodes when my level of friend-liness had deviated significantly from this personal norm. The theoretical rationale is that any increases in speed of cognizing that are leveraged by the rapid retrieval of generic self-referent trait information would be offset by limitations in the appropriateness of that information in any given situation. However, accuracy can be preserved when the boundary conditions on the validity of the generic trait information—it’s scope—are set in place by primed episodic memories of exceptions. These specific memories are slower to retrieve, thus creating an overall speed–accuracy trade-off (Klein et al., 2002).

The question we wanted to examine was why, if the system is thus optimized for accuracy, are self-relevant trait judgments routinely biased? This is the case not only in those with mental health problems, such as depression (Dalgleish & Werner-Seidler, 2014), who are characterized by biased and maladaptive views of the self, but also in healthy individuals who are characterized by biased overly positive self-views (Taylor & Brown, 1988).

We hypothesized that such biases in trait judgments may in part result from asymmetries in the aforementioned episodic memory priming effects, such that for those experiencing depression, there would be greater priming of negative episodic memories that were thus inconsistent with positive self-referent traits, than vice versa. We predicted the opposite pattern in healthy participants. We also examined performance in individuals with a history of depression who were in remission, but for whom we had no a priori predictions.

Our results using the memory priming paradigm established by Klein (Klein et al., 2001, 2002) provided support for both hypotheses. In individuals with depression, recollection of countertrait specific negative memories was relatively faster following their evaluation of a positive trait (relative to the definition control condition), compared to the other way around. This contrasted with never-depressed controls who exhibited relatively faster recollection of positive countertrait memories following evaluation of a negative self-referent trait, compared to the other way around. Individuals remitted from depression did not appear to exhibit a significant priming effect following evaluation of either positive or negative self-traits, suggesting an attenuation of the bias evident in those currently experiencing a depressive episode, but also a potential lack of reinstatement of the bias associated with stable mental health.

The present findings, as discussed in the Introduction, suggest that the relatively reduced accessibility of specific negative information necessary to set realistic constraints on the “scope” (Klein et al., 2002) of positive self-generalizations may be one potential process supporting the ubiquitous positive self-biases associated with mental health (Taylor & Armor, 1996; Taylor & Brown, 1988). In contrast, relatively “unbounded” (Klein et al., 2002) negative self-generalizations in depression may be one process that fosters, maintains, and amplifies globalized negative self-referent thinking patterns (including overgeneralization, catastrophization, negative self-attributions, disqualification of positive information), as well as perpetuating rumination (Rimes & Watkins, 2005) - factors that are known to exacerbate depression severity and persistence and that have been shown to permeate daily cognition in depression sufferers (Beck, 1967; Beck et al., 1979). Interventions designed to ameliorate disturbance in the accessibility of autobiographical memories (e.g., by improving accessibility of specific memories of positive events; cf. memory specificity training; Raes, Williams, & Hermans, 2009) may thereby help to shift the over-generalized negative thoughts and beliefs that are characteristic of depression. These interventions have shown considerable promise in alleviating depressive symptoms (for review see Hitchcock, Werner-Seidler, Blackwell, & Dalgleish. 2017) and the current results suggest that these interventions may work in part through reinstating memory retrieval patterns that help to set ‘boundaries’ on negative self-beliefs. Further exploration of this potential mechanism may help to elucidate whether interventions translated from basic science offer a low-intensity option for shifting maladaptive cognitions targeted in more complex therapies (e.g., cognitive therapy).

Although our results are consistent with the theoretical notions advanced by Klein et al. (2002), it is important to note that priming of countertrait specific memories following a trait judgment does not itself directly demonstrate that those trait generalizations have been bounded by the retrieved specific event memories. At the very least, one would want to show that trait judgments systematically differ when putative relevant boundaries are in place relative to when they are not. However, to demonstrate this a methodology that permits the manipulation of these mnemonic boundaries would be required, and it is not immediately clear what such a methodology would look like. It is also unclear whether the specific memories actually need to be explicitly retrieved as episodic recollections in order to exert any delimiting effects, or whether their primed activation is sufficient to appropriately temper the self-referent trait judgment at hand.

An interesting question is how the present findings fit with extant theories of autobiographical memory, prototypically exemplified by the framework of Conway and Pleydell-Pearce (2000). Conway and Pleydell-Pearce’s model (see also Williams et al., 2007 for an application to depression) proposes a hierarchically organized system comprising levels of representations corresponding to varieties of personal semantic information and event specific knowledge. No explicit provision is made for the sorts of priming relationships outlined by Klein et al. (2002) and supported by the present data. However, one could posit some form of tag or pointer to inconsistent event specific knowledge, contained within personal semantic memories, would provide the requisite functionality. This would concord with solutions to this issue proposed by theories with family resemblance to Klein et al.’s model (e.g., Babey et al., 1998; Graesser et al., 1979; Nosofsky et al., 1994; Schank, 1980, 1982).

Although the present study focuses on self-referred trait judgments, it seems plausible that such asymmetrical priming effects, as a potential index of systematic biases in how semantic information is applied in the world, would have relevance in other domains of cognition. Most local to the present study would be personal semantic information other than trait judgments; for example, the sorts of evaluations of the past or future that are also problematic in depression and subject to the same positive biases in the mentally healthy (e.g., Sharot, 2011). Trait judgments of others would also provide a fertile ground for investigating putative priming asymmetries (e.g., Babey et al., 1998). Klein and colleagues have demonstrated similar priming effects with respect to judgments about close others (Klein et al., 2001) and the relevance of this approach to understanding the utilization of stereotypes versus individuating information about others, and the cognitive substrates of prejudice, are intriguing (Stangor & Crandall, 2013).

In summary, the present results provide support for the notion that asymmetrical priming effects in memory may in part underpin both positive self-reference biases in the mentally healthy and negative self-referent biases in those with depression. They have implications for extant theories of autobiographical memory (e.g., Conway & Pleydell-Pearce, 2000) and potentially for novel memory-training based interventions to alleviate depression (Dalgleish & Werner-Seidler, 2014; Hitchcock et al., 2017).

## Supplementary Material

Supplemental materials: http://dx.doi.org/10.1037/xge0000343.supp

Supplemental Material

## Figures and Tables

**Table 1:** Mean (Standard Deviation) Sample Characteristics by Group

Variable	Never-Depressed (*n* = 24)	Remitted (*n* = 17)	Depressed (*n* = 16)
Age in years	28.63 (11.01)	52.11 (14.75)	49.12 (14.34)*
Number of females	14	12	11
Education	7:9:6:2	5:5:5:2	5:3:4:4
Number currently employed	9	9	9
Mill Hill Vocabulary Test	20.14 (4.52)	23.44 (5.02)	22.18 (5.39)
Verbal Fluency	42.58 (11.49)	39.50 (10.61)	37.29 (8.80)
BDI-II	3.71 (4.22)	12.13 (9.33)	27.18 (11.51)

*Note*. BDI-II = Beck Depression Inventory-Second Edition; Education = number of participants to have completed high school: undergraduate degree: postgraduate degree: diploma or professional training.

* All groups differed from one another at *p* < .001.

## Footnotes

1 It is important to note that perturbed autobiographical memory retrieval patterns (for review see Dalgleish & Werner-Seidler, 2014) are only one of many cognitive deficits associated with depression (e.g., attention, Peckham, McHugh, & Otto, 2010; processing speed, Tsourtos, Thompson, & Stough, 2002; for review of cognitive distortions in depression see Gotlib & Joormann, 2010) and that depression is associated with slower response times on a range of cognitive tasks (Azorin, Benhaïm, Hasbroucg, & Possamaï, 1995; Miller, 1975; Rogers, Bellgrove, Chiu, Mileshkin, & Bradshaw, J2004), particularly those requiring memory recall (e.g., Brand, Jolles, & Gispen-de Wied, 1992).

2 This interaction remained significant when age was included as a covariate in the analysis, *F*(2, 53) = 6.32, *p* = .003, $\eta_{p}^{2}$ = .19.
