# Supplementary material for: The Devil’s in the Detail: Accessibility of Specific Personal Memories Supports Rose-Tinted Self-Generalizations in Mental Health and Toxic Self-Generalizations in Clinical Depression
Source: J Exp Psychol Gen. Author manuscript; Available in PMC 2019 Jul 1. (PMC6600872; doi:10.1037/xge0000343)
Supplement: Supplemental Material [file EMS83500-supplement-Supplemental_Material.doc]

**Supplemental Materials**

**The Devil's in the Detail: Accessibility of Specific Personal Memories Supports Rose-Tinted Self-Generalizations in Mental Health and Toxic Self-Generalizations in Clinical Depression**

**by C. Hitchcock et al., 2017, *Journal of Experimental Psychology: General***

**http://dx.doi.org/10.1037/xge0000343**

**Data cleaning**

We examined the normality of data within each group due to expected differences in the data between never-depressed, remitted, and currently depressed groups. A positive skew was observed, as is common in response time data . This was particularly evident in the time taken to retrieve a specific memory after providing the dictionary definition of a negative word, whether the memory was consistent (never-depressed, skew = 4.02; depressed, skew = 3.35) or inconsistent (never-depressed, skew = 4.50; depressed, skew = 3.02) with the defined characteristic. The disproportionate number of extreme values in trials following a negative definition implies that long response times are a result of the experimental manipulation, not random events . Variability was also large in these conditions, expressed through large standard deviations (see Supplementary Table 1). We therefore applied a square root transformation to all data, as this transformation maintains order of values while both stabilizing variance and reducing positive skew . Transformed data were used in all analyses.

Use of response time data also requires removal of trials in which the participant responded too quickly to be plausible, as these trials represent participant error . Based on our previous experience assessing cued-recall of specific memories in both clinical and non-clinical samples , a response time of less than one second was deemed too short for a participant to retrieve a specific memory, and press the response key. Three participants in the never-depressed group, one in the remitted and one in the depressed group consistently responded in less than one second, raising concerns that they were not appropriately following task instructions. Indeed, mean response time for these participants was significantly lower in all eight experimental conditions, compared to those who did not meet our non-compliance criteria, *t*(60) > 2.10, *p*s < .040. Their data were therefore set aside from analyses. That said, it is important to note that our key hypothesized four-way interaction remained significant when non-compliers were included, *F*(2, 59) = 4.16, *p*  = .020, and also when untransformed data were used, *F*(2, 54) = 4.66, *p*  = .014, indicating that data cleaning did not impact the pattern of results. The final dataset consisted of 24 participants in the never-depressed group, 17 in the remitted group, and 16 in the depressed group.

**Integrity checks**

Prior to hypothesis testing, it was important to determine that response time would not be shorter in any specific condition simply because the cue words were more self-relevant, compared to the other conditions. For example, if words used to cue a specific memory were more self-relevant in one condition, shorter response times could simply reflect the salience or relevance of the cue word. We therefore completed valence × consistency × task interactions on the participants’ post-task self-relevance ratings for the cues used in the initial task component, and for the cues used to prompt the specific memory. These analyses were completed separately for each group, as it was likely that never-depressed individuals would rate positive characteristics as more self-relevant than negative characteristics. Indeed, this was the case for both never-depressed, *F*(1, 23) = 234.75, *p*  < .001, and remitted individuals, *F*(1, 16) = 17.57, *p*  = .001.

For never-depressed individuals, a non-significant interaction between valence, consistency and task provided no support for any difference between experimental conditions in the self-relevance of the word used in the initial component, *F*(1, 23) = 1.60, *p*  = .22, or of the word used to cue the specific memory, *F*<1. This was also the case for the remitted group, (specific memory cue; *F*<1, initial component cue; *F*<1) and the depressed group (specific memory cue; *F*<1, initial component cue, *F*<1. Thus, self-relevance of the cue is unlikely to have impacted results.

|  | Negative | | | | |  | Positive | | | | |  |
| --- | --- | --- | --- | --- | --- | --- | --- | --- | --- | --- | --- | --- |
|  | Inconsistent | |  | Consistent | |  | Inconsistent | |  | Consistent | | |
| Group | Definition | Self-rating |  | Definition | Self-rating |  | Definition | Self-rating |  | Definition | Self-rating | |
| Healthy | 12136.30  (12353.90) | 9292.50*  (5235.11) |  | 11561.65  (14105.99) | 10636.92  (7240.95) |  | 10763.88  (4649.80) | 10877.93  (5015.46) |  | 9019.83  (5001.50) | 8552.02  (4835.98) | |
|  |  |  |
| Remitted | 10257.12  (5997.04) | 10022.02  (6314.08) |  | 9673.43  (6905.96) | 11388.63  (10747.88) |  | 10006.91  (6724.45) | 9501.91  (5334.85) |  | 11094.38  (9812.59) | 11498.74  (10235.69) | |
|  |  |  |
| Depressed | 11420.68  (12625.92) | 10399.28  (7609.66) |  | 11158.61  (13978.59) | 8916.41  (6342.79) |  | 13103.54  (13796.14) | 8606.72*  (7384.80) |  | 9634.85  (7338.06) | 8844.40  (6239.49) | |
|  |  |  |

Supplementary Table 1

Untransformed mean (standard deviation) response time in milliseconds by condition

Note. * = significant difference at *p* < .05 between response time for self-rating and definition tasks. Transformed means were used in all analyses

Supplementary Table 2

Stimuli used in the Autobiographical Memory Priming Task

| Positive Cue | Antonym | Negative Cue | Antonym |
| --- | --- | --- | --- |
| adventurous | timid | afraid | calm |
| brave | cowardly | anxious | composed |
| confident | fearful | bad | good |
| determined | weak | boring | interesting |
| helpful | disagreeable | irritable | pleasant |
| hilarious | serious | naive | clever |
| hopeful | hopeless | submissive | disobedient |
| lively | gloomy | desperate | contented |
| loyal | unreliable | failure | successful |
| patient | agitated | foolish | wise |
| playful | lifeless | guilty | innocent |
| polite | impolite | helpless | capable |
| powerful | insignificant | inadequate | perfect |
| productive | unproductive | lazy | active |
| proud | sorry | mean | kind |
| resourceful | unintelligent | negligent | attentive |
| romantic | unromantic | rejected | agreeable |
| secure | insecure | rude | sophisticated |
| sincere | insincere | selfish | unselfish |
| bright | dull | snobbish | humble |
| strong | broken | stubborn | reasonable |
| talented | unskilled | temperamental | reliable |
| thoughtful | thoughtless | tense | relaxed |
| tolerant | intolerant | tired | energetic |
| trustworthy | untrustworthy | unfair | fair |
| vibrant | sluggish | vengeful | forgiving |
| warm | cold | defeated | victorious |
| able | incompetent | violent | gentle |
| admired | criticized | worried | happy |
| friendly | unfriendly | withdrawn | sociable |
| likable | unpleasant | worthless | important |
| neighborly | unsociable | pessimistic | cheerful |

*Note.* Words were randomized to experimental condition between participants.

**References**

Bargh, J. A., & Chartrand, T. L. (Eds.). (2014). *The Mind in the Middle: A Practical Guide to Priming and Automaticity Research* (2 ed.). New York, NY: Cambridge University Press.

Kenny, D. A. (1994). *Statistics for the Social and Behavioral Sciences*. New York; Harper Collins College Publishing, Incorporated.

Tabachnick, B. G., & Fidell, L. S. (2001). *Using Multivariate Statistics*. United States of America: Pearson Education.
